# Interaction between major dietary patterns and cardiorespiratory fitness on metabolic syndrome in Iranian adults: a cross-sectional study

**DOI:** 10.1186/s12937-021-00695-4

**Published:** 2021-04-13

**Authors:** Hossein Shahinfar, Mahtab Ghanbari, Yahya Jalilpiran, Nastaran Payande, Mahshid Shahavandi, Nadia Babaei, Kurosh Djafarian, Cain C. C. Clark, Sakineh Shab-Bidar

**Affiliations:** 1 Department of Community Nutrition, School of Nutritional Sciences and Dietetics, Tehran University of Medical Sciences (TUMS), No 44, Hojjat-dost Alley, Naderi St., Keshavarz Blvd, Tehran, Iran; 2 Department of Clinical Nutrition, School of Nutritional Sciences and Dietetics, Tehran University of Medical Sciences (TUMS), Tehran, Iran; 3 Centre for Sport, Exercise, and Life Sciences, Coventry University, Coventry, CV15FB UK

**Keywords:** Dietary pattern, Cardiorespiratory fitness, Metabolic syndrome, VO2max

## Abstract

**Background:**

Several researches have been conducted on the associations between diet and cardiorespiratory fitness (CRF) and major cardiovascular risk factors. However, there is no report about the interaction between major dietary patterns and CRF on metabolic syndrome (MetS) and its components. To investigate the combined association of major dietary patterns and CRF on MetS and its components.

**Methods:**

This cross-sectional study was conducted on 270 apparently healthy adults living in Tehran, Iran. Dietary intake was evaluated using a validated food frequency questionnaire (FFQ). CRF was assessed using a graded exercise treadmill test. Socio-economic status, anthropometric measures, biochemical parameters, and blood pressure were evaluated according to standard methods. Major dietary patterns were identified by factor analysis.

**Results:**

Three major identified dietary patterns were (healthy, mixed, and western). Significant positive association was found between mixed dietary pattern and metabolic syndrome (OR = 2.68, 95% CI (1.92,7.78), *P* = 0.04). There were not relations between tertiles of identified dietary patterns and remained outcomes. Those who had higher adherence to mixed pattern with also higher CRF showed a significant decrease for diastolic blood pressure (*P* < 0.01). Also we found that there was no significant interaction between any of dietary patterns and CRF on odds of MetS.

**Conclusions:**

Overall, adherence to mixed dietary pattern in this population was associated with increasing odds of MetS. However, nor CRF neither the combination of dietary patterns and CRF was related to the odds of MetS among Iranian adults. More studies are needed to clarify these associations and to consider interpersonal determinants.

**Supplementary Information:**

The online version contains supplementary material available at 10.1186/s12937-021-00695-4.

## Introduction

Metabolic syndrome (Mets) is a cluster of conditions that occur together, including excess fat accumulation around the waist, impaired metabolism of glucose due to insulin resistance, dyslipidemia such as increased blood triglycerides (TG), decreased high-density lipoprotein cholesterol (HDL-C) [1, 2]. The prevalence of metabolic syndrome components varies in different populations due to their genetic and lifestyle differences [3], But it is important to note that metabolic syndrome can predispose individuals to type II diabetes and cardiovascular disease [4, 5]. The results of several studies indicated that in addition to obesity, low cardiorespiratory fitness (CRF) is also one of the risk factors associated with metabolic syndrome. Interestingly, these studies showed that increasing amounts of physical activity and higher CRF in people contribute to their metabolic health even between obese individuals [6–10]. Cardiorespiratory fitness is also identified as a key predictor of premature death and cardiovascular disease [11–13]. CRF is defined as maximum capacity of the cardiovascular and respiratory system to supply oxygen to the skeletal muscles during exercise [5]. According to studies, CRF is positively associated with higher physical activity, lower BMI, and waist circumference in adults [14]. Lee et al., in a prospective longitudinal study of 3148 healthy adults, ages 18 or older, indicated that maintaining or improving fitness can reduce the risk of metabolic syndrome [15]. Besides, it seems that diet can be another important key to higher CRF and lower risk of Mets [14, 16]. According to previous evidence, CRF are positively associated with regular consumption of fruits, vegetables, bread and dairy products [16, 17], but the current approach of nutritional epidemiology is to research the effects of dietary patterns instead of food groups on CRF with different disease such as metabolic syndrome [18, 19]. The results of a study on cardiorespiratory fitness and healthy dietary pattern showed that fitter teenagers have healthier dietary patterns than less fitter teenagers [17]. Also, The results of a clinical trial by Neuhouser et al. conducted on 48,835 postmenopausal women showed that the low-fat dietary pattern intervention for 1 year can reduce the risk of metabolic syndrome in postmenopausal women about 17% compared to postmenopausal women with usual dietary pattern [20]. Despite the studies mentioned, limited data are available on the study of CRF-related dietary patterns in the Middle East [21]. Only limited studies have examined the interaction of various factors with metabolic syndrome and other diseases [22–25]. Also based on our search no available study has evaluated the interaction between dietary patterns and cardiorespiratory fitness on metabolic syndrome. In this study, the interactions between major dietary patterns and cardiorespiratory fitness on metabolic syndrome **in** Iranian adults were investigated.

## Methods

### Study design

In this cross-sectional study, 270 apparently healthy adults, including 118 men, and 152 women enrolled. Participants were recruited by using convenience sampling method. Based on previously calculated correlation coefficient between dietary pattern and cardiorespiratory fitness [26]. our target number of participants was 256 ((${Z}_{1-\frac{\alpha }{2}}$ $+{Z}_{1-\beta}\times \sqrt{1-{r}^2}$ /r) = 256). However, in order to replace patients who were excluded due to under- or over-reported food intakes, we continued sampling until enrolling 270 individuals.

The research criteria included an age range of 18–45, participants who apparently healthy, having desire to take part in the study, subjects who reside in Tehran. We excluded those who have extreme values of dietary intake (less than 800 kcal/d or more than 4200 kcal/d, respectively), suffering from kidney, liver, and lung diseases and other conditions affecting the body composition status or infectious and active inflammatory diseases, pregnancy, lactation, routine supplement or drug use, such as weight loss, hormonal, sedative drugs, thermogenic supplements like caffeine and green tea, conjugated linoleic acid (CLA), etc. This study was conducted according to the guidelines laid down in the Declaration of Helsinki. All necessary explanations about the project were given to the participants. All procedures were in accord with the ethical standards of the Tehran University of Medical Sciences (Ethic Number: IR.TUMS.VCR.REC.1396.4085), who approved the protocol and informed consent form. All participants signed a written informed consent prior to the start of the study.

### Assessment of other variables

Participants completed a questionnaire designed to assess the participants’ demographic including age, gender (male/female), marital status (single/married), smoking (non-smoker/former smoker/current smoker), diabetes (yes/no), cardiovascular disease (yes/no) and menopause status (yes/no). Physical activity was assessed using the international physical activity questionnaire (IPAQ) [27]. Subjects were grouped into three categories including very low (< 600 MET-minute/week), low (600–3000 MET-minute/week), moderate and high (> 3000 MET-minute/week) calculated based on Metabolic Equivalents (METs) [28].

### Dietary intakes

The dietary intake of participants was assessed by a valid and reliable semi-quantitative Food Frequency Questionnaire (FFQ), which contained 168 food items [29]. FFQ was administered by trained dietitians via face-to-face interviews, asking participants to report their frequency of consumption of each food item, during the past year on a daily, weekly, or monthly basis. To convert the portion sizes of the consumed foods to grams, household measures were used. Mean energy and nutrient intakes from the FFQs were calculated using a modified version of NUTRITIONIST IV software for Iranian foods (version 7.0; N-Squared Computing, Salem, OR, USA).

### Anthropometric measures

Body weight was determined using a standard body weight scale (seca 707; Seca GmbH & Co. KG., Hamburg, Germany). Height was measured with a tape measure mounted on the wall. Participant’s height was measured without shoes by a Stadiometer (Seca, Germany). We measured waist circumference (WC) based on the middle of bottom ribs and pelvic bones after a normal exhale using an inelastic tape. To calculate waist-hip ratio (WHR), WC in centimeter divided by hip circumference in centimeter. Body mass index (BMI) calculated as weight in kilogram divided by height in meters squared. Body composition was measured using body composition analyzer (InBody 720, Biospace, Tokyo, Japan). For this analysis, all patients were asked to follow these conditions before measurement: no food ingestion for at least 4 h, minimal intake of 2 L of water the day before, no physical activity for at least 8 h, no coffee or alcoholic beverage consumption during at least 12 h, and no diuretic use for at least 24 h. Patients were required to empty their bladder immediately before the body composition test [30]. To assess blood pressure, first we asked participants to sit for 10 min. Blood pressure was then measured using a standard mercury sphygmomanometer, twice with a 5 min interval, while participants were sitting. The mean of the two measurements was recorded as the participant’s blood pressure.

### Laboratory investigations

All participants donated 10 ml of blood between the hours of 7–10 am, in a fasted status. Following this, blood samples were collected in acid-washed test tubes without anticoagulant. Then, after being stored at room temperature for 30 min and clot formation, blood samples were centrifuged at 1500 g for 20 min. Serums were stored in – 80 °C until future testing. Fasting blood sugar (FBS) was assayed by the enzymatic (glucose oxidase) colorimetric method using a commercial kit (Pars Azmun, Tehran, Iran). Serum total cholesterol (TC) and high-density lipoprotein cholesterol (HDL-C) were measured using a cholesterol oxidase phenol aminoantipyrine method, and triglyceride (TG) was measured using a glycerol-3 phosphate oxidase phenol aminoantipyrine enzymatic method. Serum low-density lipoprotein cholesterol (LDL-C) was calculated using the Fried Ewald formula.

### Definition of terms

MetS was defined according to the National Cholesterol Education Program (NCEP) Adult Treatment Panel-III (ATP III) classification as three or more of WC > 102 cm in males and WC > 88 cm in females, fasting plasma glucose ≥110 mg/dl in both gender, or a known diagnosis diabetes, fasting serum triglyceride ≥150 mg/dl in both gender, fasting high-density lipoprotein (HDL) cholesterol < 40 mg/dl in males and HDL < 50 mg/dl in females, or blood pressure ≥ 130/85 mmHg in both gender [31].

### Cardiorespiratory fitness testing

To assess CRF, the maximum rate of oxygen consumed (VO2 max) by the treadmill, and the respiratory gas analyzer (Cortex Metabolizer 3B) was measured. Accordingly, the participants warmed up for 5 min on the treadmill at a speed of 5 km / h, and then the Bruce test was used to determine the VO2max [32]. After completing the Bruce test, the participants walked at a speed of 4 km / h in order to cool down for 3 min and perform 10 to 5 min of stretching. The conditions for the end of the test were: the patient’s heart rate reaches more than 90% of the maximum heart rate, the ratio of respiratory rate of up to 1.1, and having the plateau rate of oxygen intake, despite the increase in exercise intensity.

### Dietary patterns

Foods and beverages from the FFQ were categorized into 17 food groups based on the similarity of nutrients. Factor loadings for each of the 25 food groups were estimated using principle component analysis (PCA) method. Orthogonal transformation was used to keep identified factors uncorrelated and to improve the interpretation. Eigenvalues, the scree plot test, and interpretability were evaluated to retain factors (> 1.5) for further analysis. An absolute factor loading ≥0.3 was used to define a subset of at least 6 food groups in each factor. The identified factors were labeled based on our interpretation of the data and based on previous studies that found similar dietary patterns in adults [33–35]. Factor scores for each pattern were obtained by summing intakes of food groups weighted by their factor loadings [36]. Each participant received a factor score for each identified pattern. Participants were categorized based on tertiles of dietary pattern scores.

### Statistical analysis

All statistical analyses were performed using the Statistical Package for the Social Sciences (SPSS version 25; SPSS Inc.). We considered *p* < 0.05 as significance level. Participants were divided based on the tertiles of major dietary patterns and CRF. To compare general characteristics among tertiles, we used one-way ANOVA and chi-square tests for quantitative (height, age, fat free mas, fat mass, weight, waist circumference, waist to hip ratio, and body mass index) and qualitative variables (sex, marital status, smoking, physical activity, history of diabetes and cardiovascular disease and menopause status), respectively. Multivariate adjusted means test was performed to evaluate the association between major dietary patterns, CRF and MetS components and each of its components after adjusting for potential confounders. Two-way ANOVA was used to investigate the combined association of major dietary patterns and CRF on MetS components. Participants were categorized as dichotomously according to ATP III guideline values and first teriles were regarded as the reference group. Multivariate adjusted odds ratios test was done for indicating the interaction between CRF and dietary pattern with MetS.

## Results

**Fig. 1:**
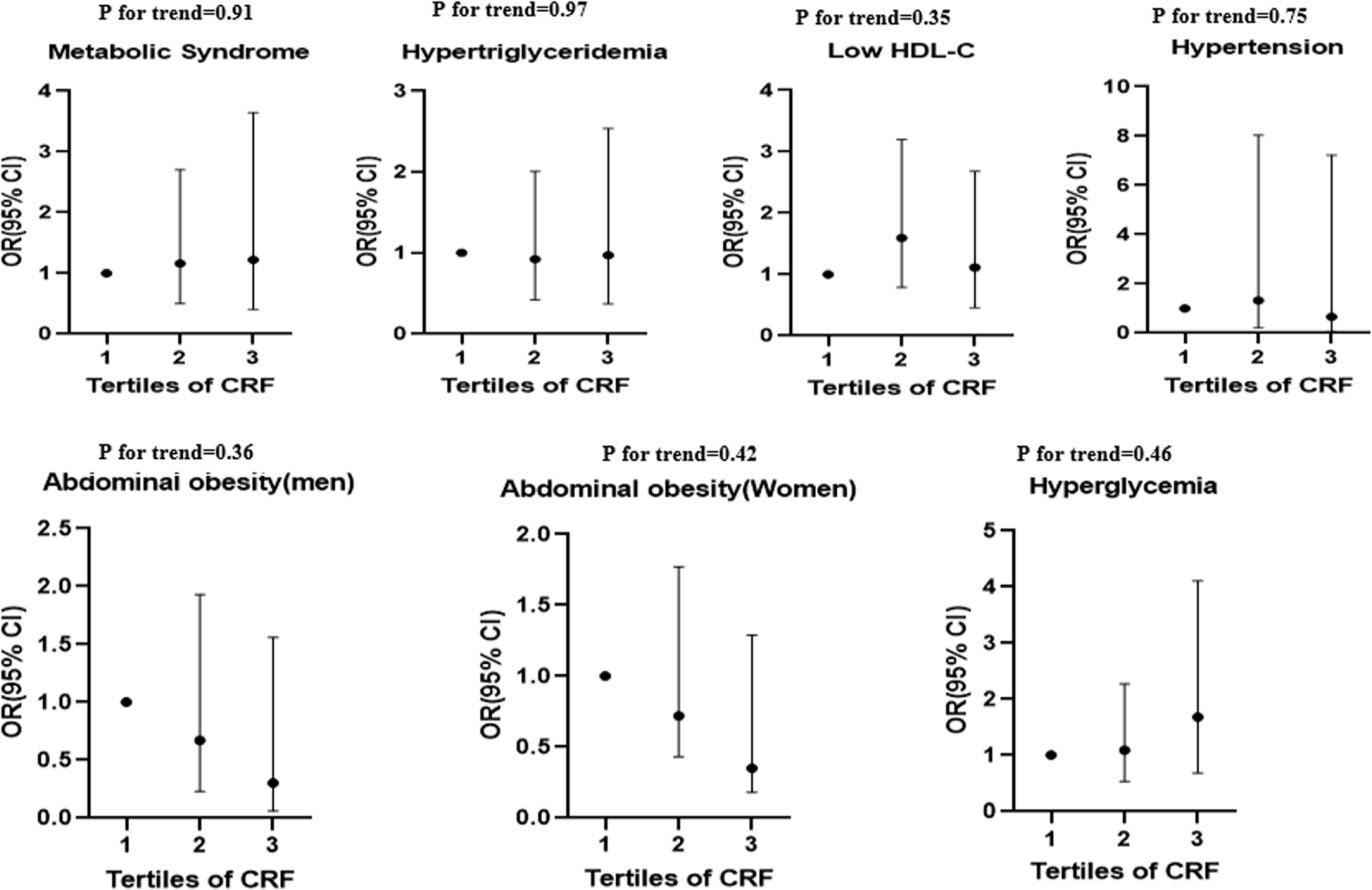
Multivariate adjusted odds ratio and 95% confidence intervals for metabolic syndrome and its components across tertiles of CRF

**Fig. 2:**
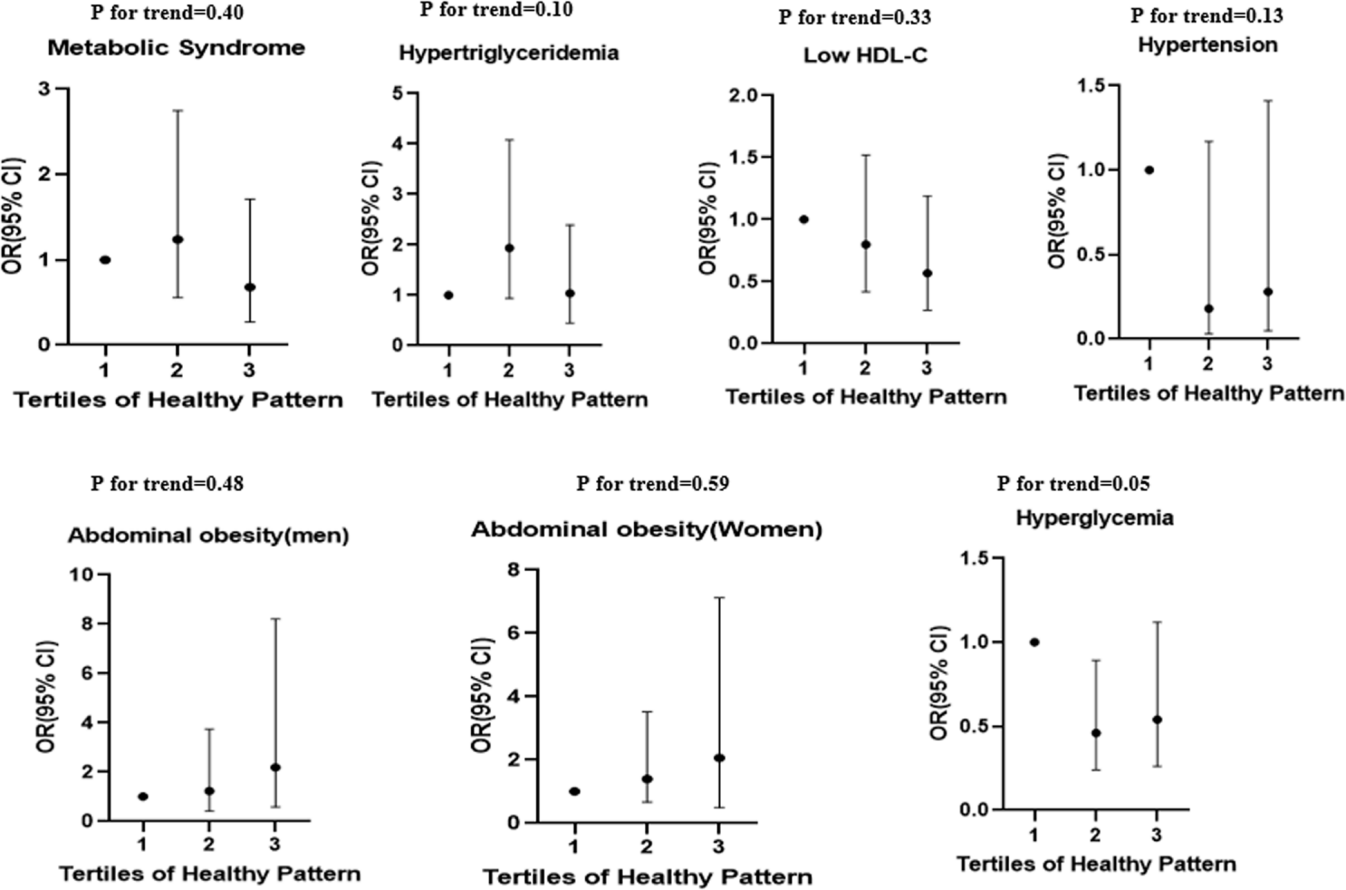
Multivariate adjusted odds ratio and 95% confidence intervals for metabolic syndrome and its components across tertiles of healthy pattern

**Fig. 3:**
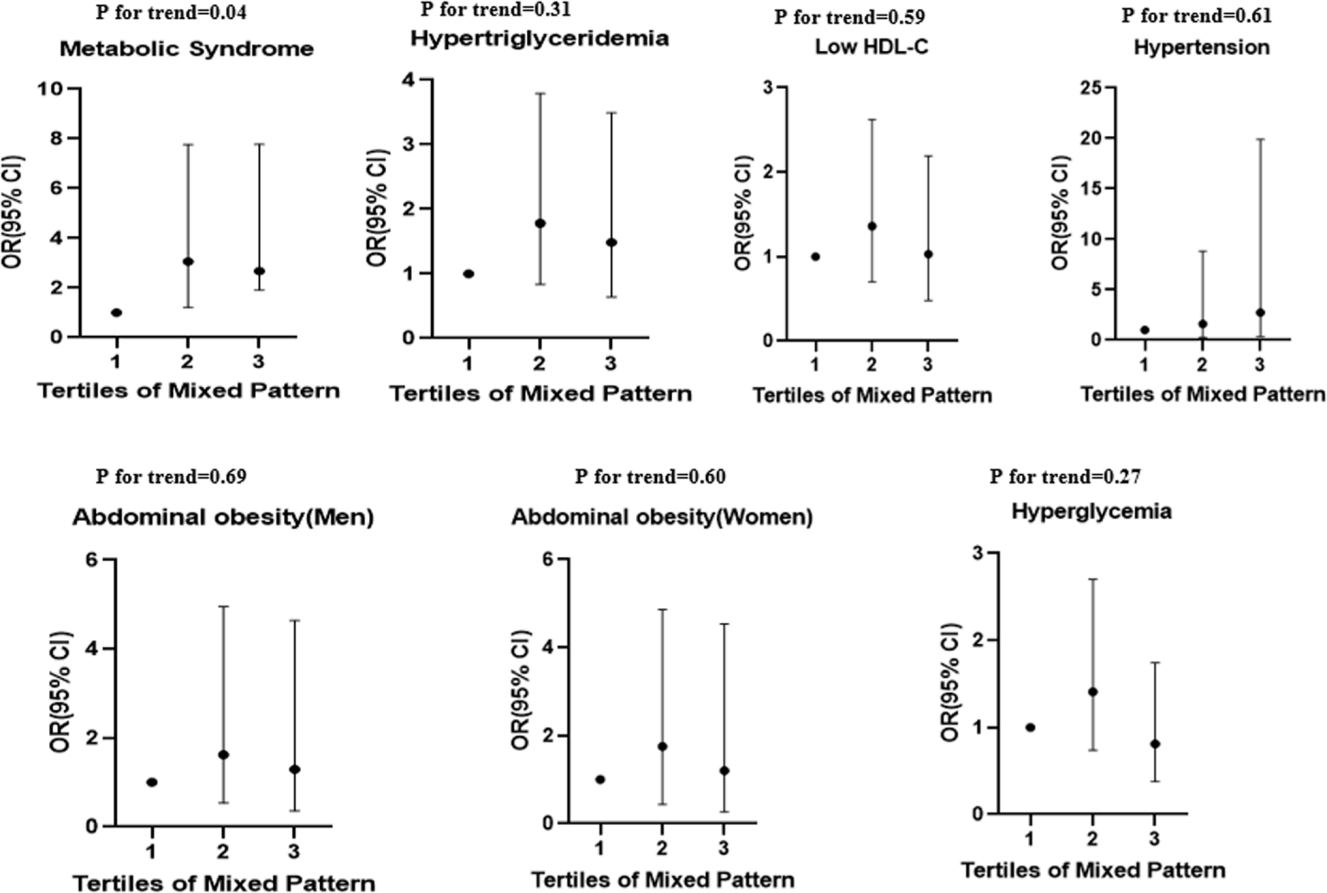
Multivariate adjusted odds ratio and 95% confidence intervals for metabolic syndrome and its components across tertiles of mixed pattern

**Fig. 4:**
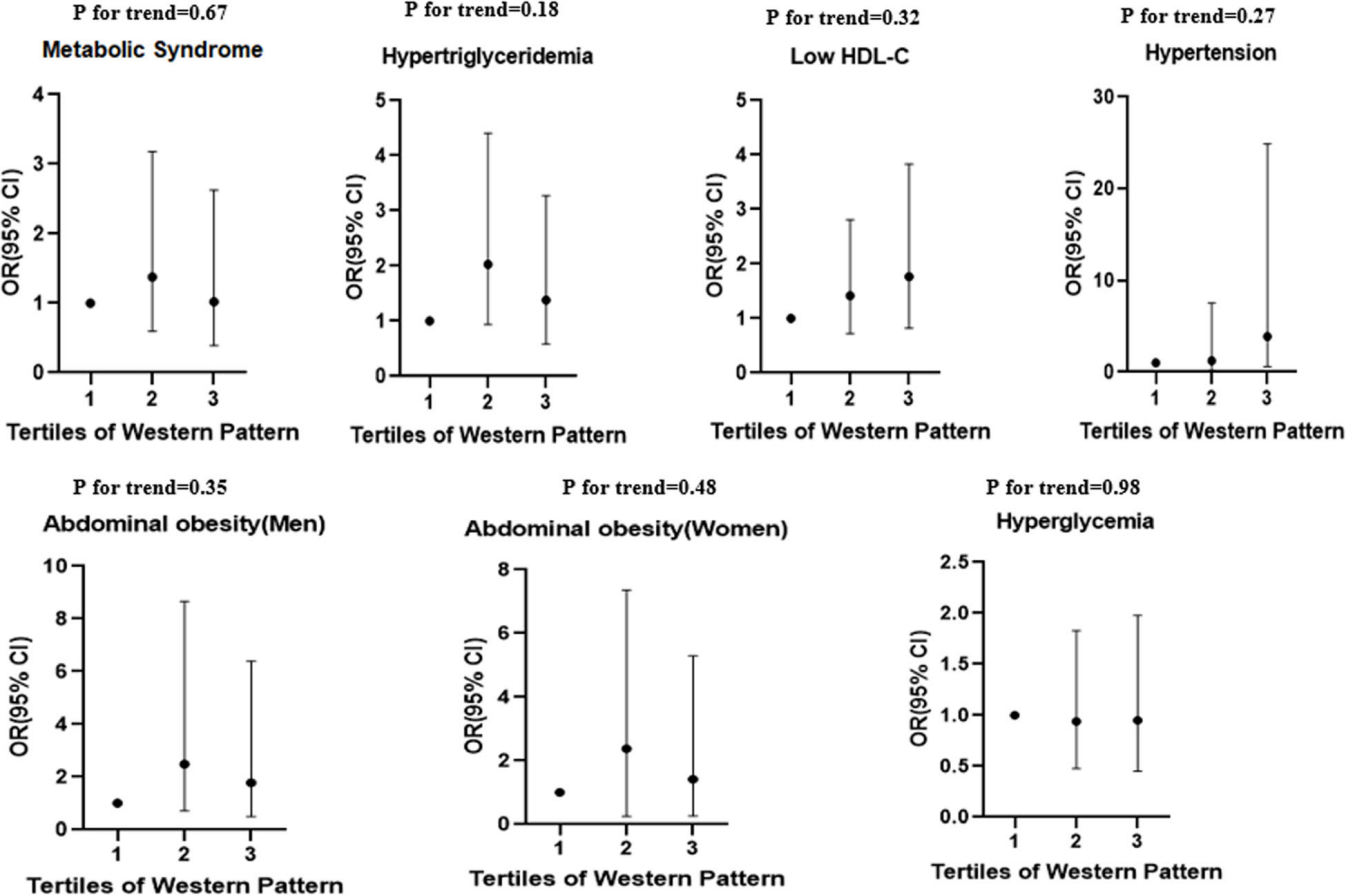
Multivariate adjusted odds ratio and 95% confidence intervals for metabolic syndrome and its components across tertiles of western pattern

Mean age of participants was 36.71 ± 13.18. The mean BMI was 25.61 ± 4.67 for them. The prevalence of abdominal obesity among men and women was 23.7 and 40.8% respectively. A total of 270 participants (117 men and 153 women) were included in this study. General characteristics of study participants by tertiles of CRF are shown in Table 1. There were also no statistical differences in distribution of smoking, and history of CVD across tertiles of CRF. General characteristics of participants across the tertiles of three major dietary patterns are indicated in Table 2**.** Participants in the highest tertile of Healthy dietary pattern (HDP) had highest BMI (*p*-value = 0.01), WC (*p*-value = 0.01). Adherence to mixed dietary pattern showed a significant increase for FM (*p*-value = 0.02), WHR(*p*-value< 0.01). Participants in the highest tertile of Western dietary pattern (WDP) had the highest FFM (*p*-value = 0.03). There were also no statistical differences in distribution of marital status, smoking and physical activity level across tertiles of all dietary patterns (Table 2). Three major dietary patterns were identified (Supplementary Fig. 1) by factor analysis which explained 34.77% of the total variance in dietary intakes amount. Table 3 shows food groups and their loading factors for three major identified dietary patterns. Positive loading demonstrated strong associations between the food groups and dietary patterns, while negative loading demonstrated negative associations **(**Table 3**)**. Multivariate adjusted means for FBS, TG, WC, HDL, SBP, and DBP across tertiles of major dietary patterns are indicated in Table 4**.** The results showed that participants in the highest tertile of WDP had the highest TG (*p* = 0.03). A significant decrease was showed for WC across the tertiles of CRF (*p*-value < 0.01). Also, Participants in the highest tertile of CRF had the highest SBP (*p*-value < 0.001), and participants in highest tertile of MDP had the lowest FBS (< 0.01). Figure 1 shows multivariate-adjusted odds ratios and 95% confidence intervals for metabolic syndrome and its components across tertiles of CRF. Multiple logistic regression models showed that there was no association between risk of metabolic syndrome and its components. Multivariate adjusted odds ratios and 95% confidence intervals for metabolic syndrome and its components across tertiles of identified dietary patterns are summarized in Figs. 2, 3, and 4. After controlling for potential covariates our results indicated significant positive association (highest vs lowest tertile) between MDP and metabolic syndrome (*P* = 0.04, OR = 2.68, 95% CI (1.92–7.78)). There were not relations between tertiles of identified dietary patterns and remained outcomes. Multivariate adjusted odds ratios and 95% confidence intervals for interaction between CRF and identified dietary patterns with metabolic syndrome are indicated in Table 5. The results showed that there was no significant interaction between CRF and HDP (*p* = 0.69), MDP (*p* = 0.80), and WDP (*p* = 0.60) with odds of metabolic syndrome after adjustment for potential confounders. Multivariate adjusted means for interaction between CRF and dietary patterns with metabolic syndrome components are shown in Table 6. Adherence to WDP showed a significant decrease for HDL across tertiles of CRF(*p*-value = 0.03). Also, adherence to HDP indicated a significant increase for SBP across tertiles of CRF (*p* = 0.03). Moreover, adherence to MDP showed a significant decrease for WC (*p*-value = 0.05) and DBP (*p*-value < 0.01) across tertiles of CRF **(**Table 6**).**

**Table 1:** General characteristics of study participants by tertiles of CRF

	All = 270 Mean ± SD or%	Tertiles of CRF			*P*_value_
		T1	T2	T3	
**n**	270	90	88	92	
**Height (cm)**	168 ± 9.96	163 ± 9.57	166 ± 49	174 ± 7.93	< 0.001
**Age (year)**	36.7 ± 13.18	42.8 ± 13.78	35.2 ± 12.36	32.1 ± 11.02	< 0.001
**FFM (kg)**	50.1 ± 12.65	46.6 ± 11.40	47.2 ± 12.68	56.3 ± 11.49	< 0.001
**FM (kg)**	22.4 ± 9.39	27.9 ± 10.23	23.2 ± 7.27	16.3 ± 6.34	< 0.001
**Weight (kg)**	72.7 ± 16.06	74.5 ± 17.82	71.0 ± 16.34	72.6 ± 13.82	0.34
**WC (cm)**					
Men	93.9 ± 12.4	105 ± 12.7	100 ± 10.8	88.4 ± 9.74	< 0.001
Women	86.3 ± 11.7	90.9 ± 12.3	83.3 ± 9.23	78.4 ± 8.21	< 0.001
**WHR**	0.90 ± 0.06	0.92 ± 0.05	0.91 ± 0.06	0.87 ± 0.06	< 0.001
**BMI (kg/m2)**	25.6 ± 4.67	27.6 ± 5.36	25.3 ± 4.12	23.8 ± 3.62	< 0.001
**Sex, male, n(%)**	117(43.5%)	16(5.9%)	32(11.9%)	69(25.7)	< 0.001
**Marital status,n(%)**					< 0.001
Single	115(43.3)	23(8.6)	36(13.5)	57(21.3)	
Married	152(56.7)	66(24.6)	51(19.0)	35(13.0)	
**Smoking, n(%)**					0.33
Non-smoker	233(86.9)	83(31.0)	75(28.0)	75(28.0)	
Former and current smoker	35(13.1)	6(2.20)	12(4.50)	17(6.30)	
**Physical activity, n(%)**					< 0.001
Low	103(38.4)	43(16.0)	39(14.6)	21(7.80)	
Medium	110(41.0)	37(13.8)	34(12.7)	39(14.6)	
high	55(20.5)	9(3.40)	14(5.20)	32(11.9)	
**Diabetes, yes, n(%)**	9(3.40)	7(2.60)	2(0.70)	0(0.0)	0.01
**CVD, yes, n(%)**	5(1.90)	3(1.10)	1(0.40)	1(0.40)	0.43
**Menopause status, yes,** **n (%)**	36(13.5)	34(12.7)	2(0.70)	0(0.0)	< 0.001

*P* value less than 0.05 was considered significant

Values are based on average ± standard deviation or reported number (percentage)

One-way anova for quantitative data and Chi-2 test for qualitative data have been used

*BMI* Body Mass Index, *WC* Waist Circumference, *FFM* fat free mass, *FM* fat mass, *WHR* waist to hip ratio, *CRF* cardiorespiratory fitness

**Table 2:** General characteristics of participants across the tertiles of three major dietary patterns

	Healthy pattern				Mixed pattern				Western pattern			
Characteristics	T1	T2	T3	*P*	T1	T2	T3	*p*	T1	T2	T3	*p*
**n**	89	90	89		89	90	89		89	90	89	
**Height (cm)**	166 ± 10.0	168 ± 9.04	169 ± 10.6	0.04	169 ± 11.0	167 ± 9.43	167 ± 9.35	0.13	165 ± 10.3	168 ± 9.70	171 ± 9.04	< 0.001
**Age (year)**	33.4 ± 12.1	35.4 ± 12.7	40.9 ± 13.1	< 0.001	32.7 ± 11.6	36.6 ± 12.5	40.3 ± 14.0	< 0.001	41.7 ± 14.4	34.0 ± 11.6	34.1 ± 11.7	< 0.001
**FFM (kg)**	47.6 ± 12.4	50.1 ± 11.6	52.7 ± 13.4	< 0.01	51.3 ± 15.0	49.3 ± 11.4	49.8 ± 11.2	0.44	48.5 ± 12.3	49.3 ± 12.2	52.6 ± 13.1	0.03
**FM (kg)**	21.6 ± 8.12	22.3 ± 9.57	23.3 ± 10.4	0.22	20.8 ± 8.17	22.4 ± 10.2	24.1 ± 9.56	0.02	22.9 ± 8.97	22.4 ± 10.5	21.9 ± 8.70	0.50
**Weight (kg)**	69.2 ± 15.8	72.5 ± 16.4	76.4 ± 15.3	< 0.01	72.6 ± 16.5	71.6 ± 16.0	73.9 ± 15.8	0.57	71.4 ± 14.0	71.7 ± 17.8	75.1 ± 16.1	0.12
**WC (cm)**												
**Men**	93.3 ± 11.4	93.8 ± 15.3	94.4 ± 10.4	0.93	93.6 ± 12.3	93.3 ± 12.7	94.7 ± 12.5	0.88	93.1 ± 10.8	94.7 ± 13.6	93.8 ± 12.6	0.87
**Women**	84.2 ± 11.4	85.9 ± 9.55	89.4 ± 13.8	0.08	83.1 ± 9.73	86.3 ± 11.8	89.2 ± 12.7	0.04	87.0 ± 10.5	84.8 ± 13.4	87.1 ± 11.0	0.53
**WHR**	0.89 ± 0.06	0.90 ± 0.06	0.91 ± 0.06	0.08	0.89 ± 0.06	0.90 ± 0.06	0.91 ± 0.07	< 0.01	0.89 ± 0.05	0.90 ± 0.07	0.91 ± 0.06	0.05
**BMI (kg/m2)**	24.8 ± 4.38	25.4 ± 4.68	26.5 ± 4.85	0.01	25.0 ± 4.12	25.5 ± 4.98	26.2 ± 4.87	0.08	26.0 ± 25.2	25.2 ± 5.35	25.5 ± 4.29	0.38
**Sex, male, n(%)**	30 (11.2)	40 (14.9)	47 (17.5)	0.03	42 (15.7)	33 (12.3)	42 (15.7)	0.26	42 (10.4)	33 (13.1)	42 (20.1)	< 0.001
**Marital status,n(%)**				0.40				0.27				0.14
Single	51 (19.0)	39 (14.6)	36 (13.4)		46 (17.2)	38 (14.2)	32 (11.9)		32 (11.9)	42 (15.7)	42 (15.7)	
Married	38 (14.2)	51 (19.0)	53 (19.8)		43 (16.0)	52 (19.4)	57 (21.3)		57 (21.3)	48 (17.9)	48 (17.9)	
**Smoking, n(%)**				0.31				0.13				0.24
Non-smoker	81 (30.2)	78 (29.1)	73 (27.2)		77 (28.7)	81 (30.2)	74 (27.6)		77 (28.7)	84 (31.3)	71 (26.5)	
Former and current smoker	8 (3.2)	12 (4.8)	16 (6.4)		12 (4.5)	9 (3.4)	15 (5.6)		12 (4.5)	6 (2.3)	18 (6.7)	
**Physical activity,n(%)**				0.10				0.19				0.92
Low	40 (14.9)	35 (13.1)	27 (10.1)		33 (12.3)	40 (14.9)	29 (10.8)		31 (11.6)	35 (13.1)	36 (13.4)	
Medium	36 (13.4)	39 (14.6)	36 (13.4)		33 (12.3)	38 (14.2)	40 (14.9)		40 (14.9)	37 (13.8)	34 (12.7)	
high	13 (4.9)	16 (6.0)	26 (9.7)		23 (8.6)	12 (4.5)	20 (7.5)		18 (6.7)	18 (6.7)	19 (7.1)	
**Diabetes, yes, n(%)**	3 (1.1)	2 (0.7)	4 (1.5)	0.70	1 (0.4)	3 (1.1)	5 (1.9)	0.25	5 (1.9)	2 (0.7)	2 (0.7)	0.35
**CVD, yes, n(%)**	2 (0.7)	2 (0.7)	2 (0.7)	0.90	0 (0.0)	3 (1.1)	3 (1.1)	0.22	2 (0.7)	1 (0.4)	3 (1.1)	0.60
**Menopause status, yes, n (%)**	11 (4.1)	10 (3.7)	15 (5.6)	0.03	6 (2.2)	12 (4.5)	18 (6.7)	0.02	21 (7.9)	10 (3.7)	5 (1.9)	< 0.001

*P* value less than 0.05 was considered significant

Values are based on average ± standard deviation or reported number (percentage)

One-way ANOVA for quantitative data and Chi-2 test for qualitative data have been used

*BMI* body mass index, *CVD* cardiovascular disease, *WC* waist circumference, *WHR* waist to hip ratio, *FFM* fat free mass, *FM* fat mass;

**Table 3:** Food groups and their loading factors for three identified dietary patterns

	Group details	Dietary Patterns		
		Healthy pattern	Mixed pattern	Western pattern
Organ meats	Heart, kidney, liver, tongue, brain, offal, rennet			
Salt	Salt			
Potato	Potato			
Hydrogenated fats	Hydrogenated vegetable oils, solid fats (animal origin), animal butter, margarine			
Poultry	Chicken	0.727		
Egg	Egg	0.648		
Legumes	Lentils, split pea, beans, chick pea, fava bean, soy, others	0.623		
Fruits and Juices	Melon, watermelon, honeydew melon, plums, prunes, apples, cherries, sour cherries, peaches, nectarine, pear, fig, date, grapes, kiwi, pomegranate, strawberry, banana, persimmon, berry, pineapple, oranges, dried fruits, all juices, others	0.582		
Fishes	All fish types	0.581		
Nuts	Almonds, peanut, walnut, pistachio, hazelnut, seeds, others	0.485		
Olive and olive oil	Olive and olive oil	0.453		
Low fat dairy product	Low-fat milk, skim milk, low-fat yogurt, cheese, *Kashk*, yogurt drink, others	0.451		
Mayonnaise	Mayonnaise		0.767	
Pickles			0.718	
High fat dairy product	High-fat milk, high-fat yogurt, cream cheese, cream, dairy fat, ice cream, others		0.543	
Non-refined cereals	Dark breads (e.g., *barbari*, *sangak*, *taftun*), bran breads, others		0.468	
Vegetables	Cauliflower, carrot, tomato and its products, spinach, lettuce, cucumber, eggplant, onion, greens, green bean, green pea, squash, mushroom, pepper, corn, garlic, turnip, others	0.538	0.467	
Vegetable oils	Vegetable oils (except for olive oils)		0.428	
Soft drinks	Soft drinks			0.699
French fries	French fries			0.621
Salty snacks	Corn puffs, crackers, potato chips, others			0.547
Sweets and desserts	Cookies, cakes, biscuits, muffins, pies, chocolates, honey, jam, sugar cubes, sugar, candies, sweet tahini, others			0.469
Refined cereals	Lavash bread, baguette bread, rice, pasta, others			0.456
Red and processed meats	Beef and veal, lamb, minced meat, sausage, deli meat, hamburger			0.401
Tea and coffee	Tea and coffee			0.313

Factor loadings < 0.03 for all three patterns were excluded

**Table 4:** Multivariate adjusted means for FBS, TG, WC, HDL, SBP, and DBP across tertiles of major dietary patterns and CRF

	Tertiles of major dietary patterns					
	Healthy Dietary Pattern					
	T1	T2	T3	P*	P _Trend_	P$
**FBS (mg/dl)**	98.5 ± 12.0	96.7 ± 10.5	99.9 ± 28.6	0.53	0.63	0.72
**TG (mg/dl)**	117 ± 65.5	132 ± 80.5	109 ± 59.8	0.08	0.46	0.10
**HDL (mg/dl)**	49.1 ± 10.5	50.8 ± 11.7	189 ± 40.2	0.47	0.98	0.50
**SBP (mmHg)**	106 ± 26.9	110 ± 10.8	116 ± 14.5	< 0.01	< 0.001	0.15
**DBP (mmHg)**	69.9 ± 11.7	69.7 ± 10.4	72.2 ± 9.75	0.22	0.15	0.72
**WC (cm)**						
Men	93.3 ± 11.4	93.8 ± 15.3	94.4 ± 10.4	0.93	0.72	0.07
Women	84.2 ± 11.4	85.9 ± 9.55	89.4 ± 13.8	0.08	0.02	0.08
	Mixed Dietary Pattern					
	T1	T2	T3	*P*	P _Trend_	P$
**FBS (mg/dl)**	101 ± 28.6	97.98 ± 10.29	96.2 ± 11.5	0.23	0.09	< 0.01
**TG (mg/dl)**	116 ± 69.7	124.90 ± 72.44	117 ± 67.1	0.70	0.92	0.81
**HDL (mg/dl)**	49.5 ± 9.81	50.54 ± 11.97	49.1 ± 10.2	0.67	0.82	0.55
**SBP (mmHg)**	109 ± 18.6	110.75 ± 20.74	114 ± 17.8	0.22	0.09	0.94
**DBP (mmHg)**	69.7 ± 8.31	69.23 ± 12.38	72.9 ± 10.7	0.04	0.04	0.63
**WC (cm)**						
Men	93.6 ± 12.3	93.3 ± 12.7	94.7 ± 12.5	0.88	0.68	0.14
Women	83.1 ± 9.73	86.3 ± 11.8	89.2 ± 12.7	0.04	0.01	0.61
	Western Dietary Pattern					
	T1	T2	T3	*P*	P _Trend_	P$
**FBS (mg/dl)**	97.9 ± 8.81	97.2 ± 12.6	100 ± 28.8	0.56	0.46	0.80
**TG (mg/dl)**	108 ± 54.90	129 ± 75.1	122 ± 75.9	0.10	0.17	0.03
**HDL (mg/dl)**	49.5 ± 9.84	50.3 ± 11.5	49.3 ± 10.7	0.80	0.52	0.86
**SBP (mmHg)**	114 ± 18.04	110 ± 19.7	109 ± 19.5	0.24	0.13	0.25
**DBP (mmHg)**	70.0 ± 13.11	70.2 ± 8.81	71.5 ± 9.80	0.59	0.35	0.17
**WC (cm)**						
Men	93.1 ± 10.8	94.7 ± 13.6	93.8 ± 12.6	0.87	0.83	0.61
Women	87.0 ± 10.5	84.8 ± 13.4	87.1 ± 11.0	0.53	0.96	0.80
	Cardiorespiratory fitness (ml/kg/min)					
	T1	T2	T3	*P*	P _Trend_	P$
**FBS (mg/dl)**	97.5 ± 9.76	100 ± 27.5	97.5 ± 14.9	0.58	0.98	0.59
**TG (mg/dl)**	123 ± 60.7	125 ± 80.2	110 ± 66.4	0.27	0.20	0.61
**HDL (mg/dl)**	49.8 ± 9.16	49.5 ± 12.0	49.9 ± 10.7	0.95	0.93	0.96
**SBP (mmHg)**	108 ± 27.9	111 ± 13.76	114 ± 11.1	0.12	0.04	< 0.001
**DBP (mmHg)**	71.1 ± 12.2	70.2 ± 11.7	70.3 ± 7.69	0.81	0.59	0.62
**WC (cm)**						
Men	105 ± 12.7	100 ± 10.8	88.4 ± 9.74	< 0.001	< 0.001	0.52
Women	90.9 ± 12.3	83.3 ± 9.23	78.4 ± 8.21	< 0.001	< 0.001	0.45

Values are presented as mean ± SD

*Abbreviations*: *FBS* Fasting Blood Sugar, *TG* Triglyceride, *HDL* High Density Lipoprotein, *SBP* Systolic Blood Pressure, *DBP* Diastolic Blood, *WC* Waist Circumference

*obtained using one-way ANOVA test

$ obtained using analysis of covariance (ANCOVA) test (adjusted for age, sex, marital status, physical activity, smoking, total energy, diabetes, cardiovascular disease history, menopause status, and body mass index

**Table 5:** Multivariate adjusted odds ratios and 95% confidence intervals for interaction between CRF and dietary pattern with metabolic syndrome

**CRF**	Tertiles of healthy dietary pattern			
	T1	T2	T3	*P*_value for interaction_
**T1**	1.00	1.00	1.00	0.69
**T2**	1.00	0.85(0.14,5.06)	1.85(0.29,11.70)	
**T3**	1.00	0.57(0.09,3.53)	0.43(0.05,3.22)	
**CRF**	Tertiles of mixed dietary pattern			
	T1	T2	T3	*P*_value for interaction_
**T1**	1.00	1.00	1.00	0.80
**T2**	1.00	1.54(0.19,12.07)	1.32(0.16,10.48)	
**T3**	1.00	1.36(0.17,10.91)	0.47(0.05,4.28)	
**CRF**	Tertiles of western dietary pattern			
	T1	T2	T3	*P*_value for interaction_
**T1**	1.00	1.00	1.00	0.60
**T2**	1.00	0.58(0.09,3.54)	1.60(0.25,10.21)	
**T3**	1.00	1.36(0.21,8.72)	0.76(0.09,6.23)	

Data are presented as OR (95%CI)

*CRF* cardiorespiratory fitness

**Table 6:** Multivariate adjusted means for interaction between CRF and dietary patterns with metabolic syndrome components

**FBS (mg/dl)**	CRF (ml/kg/min)			
Tertiles of healthy pattern	T1	T2	T3	*P*_value_
T1	98.0 ± 11.8	100 ± 14.0	96.4 ± 9.63	0.45
T2	97.6 ± 7.25	94.6 ± 8.09	98.1 ± 14.5	
T3	96.8 ± 9.58	106 ± 48.1	97.6 ± 18.1	
**FBS (mg/dl)**	CRF (ml/kg/min)			
Tertiles of mixed pattern	T1	T2	T3	*P*_value_
T1	97.5 ± 13.4	104.5 ± 42.0	99.3 ± 16.8	0.86
T2	98.3 ± 9.85	97.8 ± 10.3	97.7 ± 11.0	
T3	96.6 ± 7.41	96.6 ± 10.7	95.1 ± 15.9	
**FBS (mg/dl)**	CRF (ml/kg/min)			
Tertiles of western pattern	T1	T2	T3	*P*_value_
T1	98.7 ± 7.94	97.9 ± 8.05	96.7 ± 10.6	0.22
T2	97.1 ± 9.72	95.5 ± 13.0	99.1 ± 14.7	
T3	96.0 ± 12.8	106 ± 44.0	96.8 ± 17.8	
**TG (mg/dl)**	CRF (ml/kg/min)			
Tertiles of healthy pattern	T1	T2	T3	*P*_value_
T1	110 ± 47.2	127 ± 80.6	114 ± 70.8	0.66
T2	138 ± 65.7	133 ± 90.5	125 ± 83.3	
T3	126 ± 70.7	115 ± 66.2	94.1 ± 41.4	
**TG (mg/dl)**	CRF (ml/kg/min)			
Tertiles of mixed pattern	T1	T2	T3	*P*_value_
T1	129 ± 87.9	109 ± 64.0	117 ± 64.0	0.27
T2	118 ± 46.0	146 ± 86.4	110 ± 79.1	
T3	125 ± 56.4	126 ± 90.8	101 ± 56.4	
**TG (mg/dl)**	CRF (ml/kg/min)			
Tertiles of western pattern	T1	T2	T3	*P*_value_
T1	109 ± 45.6	121 ± 63.5	93.9 ± 57.3	0.84
T2	135 ± 63.4	136.6 ± 91.4	115 ± 65.3	
T3	133 ± 77.5	119 ± 81.0	117 ± 73.1	
**HDL (mg/dl)**	CRF (ml/kg/min)			
Tertiles of healthy pattern	T1	T2	T3	*P*_value_
T1	49.5 ± 8.56	47.1 ± 11.0	50.9 ± 12.6	0.39
T2	51.5 ± 10.0	49.7 ± 13.4	51.5 ± 11.4	
T3	48.3 ± 9.38	51.7 ± 11.5	48.0 ± 8.66	
**HDL (mg/dl)**	CRF (ml/kg/min)			
Tertiles of mixed pattern	T1	T2	T3	*P*_value_
T1	50.3 ± 9.26	49.1 ± 10.0	49.4 ± 10.1	0.65
T2	51.6 ± 9.55	48.7 ± 13.9	51.1 ± 12.5	
T3	47.7 ± 8.80	50.8 ± 12.7	49.5 ± 9.99	
**HDL (mg/dl)**	CRF (ml/kg/min)			
Tertiles of western pattern	T1	T2	T3	*P*_value_
T1	51.9 ± 9.73	47.6 ± 11.2	48.0 ± 8.19	0.03
T2	47.8 ± 7.80	49.0 ± 13.2	54.2 ± 11.8	
T3	48.6 ± 9.76	51.3 ± 11.6	48.0 ± 10.8	
**WC (cm)**	CRF (ml/kg/min)			
Tertiles of healthy pattern	T1	T2	T3	*P*_value_
T1	90.3 ± 13.8	86.0 ± 11.0	84.1 ± 10.4	0.78
T2	94.5 ± 13.6	88.9 ± 12.9	85.5 ± 11.2	
T3	96.3 ± 12.9	94.5 ± 13.4	87.3 ± 9.47	
**WC (cm)**	CRF (ml/kg/min)			
Tertiles of mixed pattern	T1	T2	T3	*P*_value_
T1	92.0 ± 15.2	88.7 ± 12.3	85.1 ± 9.35	0.05
T2	89.2 ± 13.8	88.7 ± 13.6	88.7 ± 10.0	
T3	98.0 ± 11.2	91.9 ± 12.6	84.1 ± 11.3	
**WC (cm)**	CRF (ml/kg/min)			
Tertiles of western pattern	T1	T2	T3	*P*_value_
T1	91.8 ± 11.7	86.4 ± 10.7	87.0 ± 9.26	0.32
T2	91.6 ± 15.6	90.7 ± 15.0	83.5 ± 10.4	
T3	98.3 ± 13.2	90.9 ± 11.6	86.9 ± 10.8	
**SBP (mmHg)**	CRF (ml/kg/min)			
Tertiles of healthy pattern	T1	T2	T3	*P*_value_
T1	99.0 ± 39.0	108 ± 11.9	115 ± 13.4	0.03
T2	113.0 ± 13.4	108 ± 9.23	111 ± 9.71	
T3	115.7 ± 16.9	119 ± 17.1	115 ± 10.4	
**SBP (mmHg)**	CRF (ml/kg/min)			
Tertiles of mixed pattern	T1	T2	T3	*P*_value_
T1	99.3 ± 32.6	111 ± 9.88	113 ± 11.6	0.12
T2	108 ± 28.7	107 ± 15.6	117 ± 11.3	
T3	113 ± 24.0	117 ± 14.8	112 ± 10.1	
**SBP (mmHg)**	CRF (ml/kg/min)			
Tertiles of western pattern	T1	T2	T3	*P*_value_
T1	114 ± 24.2	110 ± 11.7	117 ± 10.9	0.26
T2	106 ± 29.7	112 ± 14.9	111 ± 10.3	
T3	100 ± 31.2	111 ± 14.2	114 ± 11.3	
**DBP (mmHg)**	CRF (ml/kg/min)			
Tertiles of healthy pattern	T1	T2	T3	*P*_value_
T1	69.8 ± 14.1	69.8 ± 10.9	69.7 ± 9.06	0.78
T2	70.2 ± 10.1	68.5 ± 13.3	70.5 ± 6.84	
T3	74.0 ± 11.6	72.7 ± 10.4	70.5 ± 7.59	
**DBP (mmHg)**	CRF (ml/kg/min)			
Tertiles of mixed pattern	T1	T2	T3	*P*_value_
T1	69.0 ± 9.13	69.4 ± 9.60	70.5 ± 6.46	< 0.01
T2	68.0 ± 13.6	66.8 ± 14.5	73.1 ± 6.43	
T3	75.4 ± 11.5	75.5 ± 9.09	67.4 ± 9.21	
**DBP (mmHg)**	CRF (ml/kg/min)			
Tertiles of western pattern	T1	T2	T3	*P*_value_
T1	70.9 ± 15.1	66.0 ± 14.7	72.5 ± 6.56	0.13
T2	71.5 ± 9.16	71.1 ± 9.75	67.9 ± 6.98	
T3	71.2 ± 10.8	72.6 ± 10.5	70.6 ± 8.62	

*P* value less than 0.05 was considered significant

Data are presented as mean ± standard deviation

*P*_value_ derived from two-way analysis of variances

*CRF* cardiorespiratory fitness, *FBS* Fasting Blood Sugar, *TG* Triglyceride, *HDL* High Density Lipoprotein, *SBP* Systolic Blood Pressure, *DBP* Diastolic Blood, *WC* Waist Circumference, *mg/dl* miligram per deciliter, *cm*^*2*^ centimeter^2^, *mmHg* millimetre of mercury, *ml/kg/min* milliliter per kilogram per minute

## Discussion

Previous researches have centralized mainly on the independent effects of dietary pattern and CRF on MetS risk. In this article, in addition to examining the independent effect of dietary pattern and CRF on MetS, we have evaluated the combined association between dietary pattern, CRF, and MetS. In this cross-sectional study, we identified three major dietary patterns including healthy, mixed, and western patterns. HDP and WDP had no statistically significant association with metabolic syndrome. However, there is a statistically significant between MDP and metabolic syndrome. We found having a higher score on the CRF compared with a lower score was associated with decreased odds of MetS, however, were not statistically significant. In addition, we found that there was no interaction of any dietary patterns (healthy, mixed, and western pattern) and CRF on odds of MetS. However, Adherence to WDP showed a significant decrease for HDL across tertiles of CRF. Adherence to HDP indicated a significant increase for SBP across tertiles of CRF and adherence to MDP showed a significant decrease for WC and DBP across tertiles of CRF.

To date, several epidemiological studies have been performed on the impact of dietary pattern on MetS. Evidence from a cross-sectional study of 4984 women aged 30–79 years indicated that HDP (high in green-yellow vegetables, healthy-protein foods, seaweeds, and bonefish) is associated with a decreased risk for MetS [37]. In another study by Esmaillzadeh et al., in 486 Tehrani female teachers the HDP (high in fruits, poultry, and vegetables) was associated with less prevalence of the MetS [34]. Williams et al., in a cross-sectional study of 802 UK-population aged 40–65 years reported that a HDP (high in fruits, raw and salad vegetables, fish, and low in fried foods) is associated with decreased central obesity and fasting glucose concentration [38]. In addition, a previous prospective study of 15,972 white and black men and women 45 to 64 years of age revealed adherence to prudent dietary pattern that described by intakes of cruciferous and carotenoid vegetables, fruit, fish, and poultry, was not correlated with an increase in the prevalence of MetS [39]. In the current study, it seems that a HDP (high in legumes, vegetables, poultry, fruits and fruits juices, nuts, fish, egg, low-fat dairy product, olive, and olive oil) is neither associated with a reduction in the prevalence of metabolic syndrome nor its components. In the present study, other factors such as weight control in people who have a HDP may affect the desired effects. For example, in our study, participants in the highest tertile of HDP had highest BMI, weight, and WC. Besides, participants in the highest tertile of HDP are older than those in the lowest tertile. The prevalence of metabolic syndrome increases with age, so this may be influential. Generally, although the HDP identified in our study is rich in foods that have the potential to reduce the risk of metabolic syndrome but it cannot be propounded exclusively a health-promoting diet. This pattern is more a reflection of the effort to choose healthy foods.

WDP that is determined by excessive consumption of fat, sweets, and refined grains showed inconsistent results with metabolic syndrome and its components [40–42]. In research by Cho et al., the WDP (high in fast foods, animal fat-rich foods, fried foods, grilled meat and seafoods, and sweet foods) indicated no association with MetS [37]. However, WDP in 486 Tehrani female teachers in the study of Esmaillzadeh et al., was related to greater risks of MetS [34]. Adherence to a WDP that determined by high consumption of refined grains, processed meat, fried foods, and red meat, was correlated with an 18% higher risk of occurrence MetS [43]. In our study, Dietary consumption of a WDP (including refined cereals, red or processed meat, soft drinks, sweets and desserts, tea and coffee, salty snacks, and french fries) was not correlated with an increased risk of metabolic syndrome and its components. Perhaps one of the reasons for this conclusion is the menopausal status in WDP tertiles. As state in results, Participants in the top tertile of WDP had less menopause status. Evidence from several studies suggested postmenopausal status is correlated with an increased risk of the metabolic syndrome independent of age [44, 45]. In this regard reported the menopausal transition is linked with an increase in abdominal adiposity, independent of age and total body adiposity [46].

In relation to the MDP, which has a combination of healthy (non-refined cereals, vegetables, vegetable oils) and unhealthy (mayonnaise, high-fat dairy product) foods in present study is almost identical to the pattern described in Esmaillzadeh and colleagues study as the traditional food pattern [34]. It has been suggested that healthy combinations of this pattern can have a protective effect on the syndrome, while unhealthy foods can have a negative effect on the prevalence of the syndrome. As well as, the synergistic effect of other food compounds on HDP may be a factor in reducing the risk of metabolic syndrome [34]. The result of our analysis showed Dietary consumption of a MDP (high consumption of non-refined cereals, vegetable oils, mayonnaise, high-fat dairy product, and pickles) was not associated with odds of metabolic syndrome or its components. Anthropometric characteristics and also menopause status may influence the relationship between the MDP and the metabolic syndrome. In this pattern subjects in the highest tertile of MDP had highest FM, WC, WHR, and menopause status. Several studies have reported an opposite association between metabolic syndrome and the amount of body fat [47–49] and menopausal status [50, 51].

Our results indicated that having a higher score on the CRF compared with a lower score was associated with decreased odds of MetS, however, were not statistically significant. Also, higher CRF levels were associated with lower measures of abdominal obesity. Several studies have investigated the associations of CRF and MetS. Shaibi et al. indicated that CRF was not associated with any individual risk factor of the metabolic syndrome [52]. In addition, a population-based cohort on 605 middle-aged men and women found no independent association between CRF and the development of MetS [53]. In contrast, Ekelund et al. reported significant reverse association between CRF and with indicators of insulin resistance, hyperglycemia, hyperlipidemia, and clustered metabolic risk in children [54]. Other studies have shown that CRF is linked to insulin sensitivity as a risk factor for MetS [55, 56]. Hassinen et al., in 1226 men and women aged 57–78 years, reported that higher levels of CRF protect against MetS [9]. Increased muscle insulin sensitivity, lipoprotein lipase activity in active musculoskeletal, transport of lipids and lipoproteins from the peripheral blood circulation and tissues to the liver, and reduction abdominal obesity [57] are mechanisms by which MetS may improve with fitness [58, 59]. The differences between the results of the association between CRF and MetS in different studies may be related to different sample sizes, group characteristics, and differences in study methods. In addition, genetic, age, body composition, and most importantly, physical activity determines CRF [60]. In this study, subjects who have top levels of CRF have more physical activity, have greater FFM, lower BMI, WC, and WHR than those who have lower level of CRF. Indeed, CRF moderates the link between obesity and metabolic status [61]; considering that the risk of metabolic disorders, including MetS, increases with obesity.

All of these articles separately evaluated the association between dietary pattern and cardiorespiratory fitness on metabolic syndrome. To our knowledge, just three previous studies have examined the combined association between the dietary pattern and CRF with MetS. Kouki et al., in a sample of 663 men and 671 women 57–78 years of age revealed healthy diet, and higher levels of CRF are associated with a reduced risk of having MetS [62]. A cross-sectional school-based study was conducted on 468 adolescents aged 15**–**18, suggested the chances of having MetS are significantly higher among adolescents with low adherence to the Southern European Atlantic Diet (SEADiet) which has emerged as a healthy dietary pattern compared with adolescents with higher adherence to SEADiet [63]. Liao et al., in a cross-sectional study among 615 children (354 boys and 261 girls) aged 9.6 ± 0.6 years reported a significantly interactive relationship between high CRF and healthy dietary intake with metabolic health among Chinese children [64]. The results of these studies suggested that a combination of diet and CRF may have synergistic effects on metabolic health. Results from the current study showed that there was no significant interaction between CRF and HDP, MDP, and WDP with odds of MetS. The conflicting results between our study and the studies mentioned above may be due to differences in socio-demographic characteristics, ethnicity, behavioral and lifestyle factors, differences in study population, and differences in dietary data collection methods.

In this study, follow to HDP showed a significant increase for systolic blood pressure across tertiles of CRF. Interestingly, some people develop high blood pressure notwithstanding an active physical lifestyle or a healthy diet, while some sedentary people with unhealthy dietary patterns may have optimal blood pressure. A number of studies have shown that higher cardiorespiratory fitness and physical activity are associated with lower blood pressure [65, 66]. However, some studies did not find a connection [67] and in some cases, it is even accompanied by a scant increase [60]. The two chief factors that can lead to these differences are the level of primary phenotype and familial aggregation [60]. In general, information on the contribution of genetic diversity to the antihypertensive effect associated with cardiorespiratory fitness is low. In this study, more adherence to the MDP was associated with an increase in waist circumference and blood pressure. But in people who have a MDP, waist circumference and diastolic blood pressure decreased with increasing physical activity and cardiorespiratory fitness. Physical activity decreased the effect of genetic factors like FTO genotype to cause high BMI and WC [68]. Eventually, high cardiorespiratory fitness overcame the harmful effects of a mixed dietary pattern on WC and DBP. In relation to the interaction of western dietary pattern and cardiovascular fitness with the components of MetS it is notable that, despite have reported that HDL-C is highly responsive in exercise and have ascertained that exercise and high level of cardiorespiratory fitness increase HDL-cholesterol [69] adherence to WDP showed a significant decrease for HDL across tertiles of CRF. In many studies, the western dietary pattern was inversely associated with HDL-C [70, 71]. One mechanism clarifying the association between the WDP and lower HDL-C could be higher intake of refined carbohydrates among participants who follow the WDP [71]. Finally, western dietary pattern dominates the detrimental effects of a cardiorespiratory fitness on HDL-C.

Our study had some strengths. According to our knowledge, that interaction between three major dietary patterns and cardiorespiratory fitness on metabolic syndrome is reported for the first time in our study. We used a 168-item questionnaire and reduced the potential for residual confusion by collecting comprehensive dietary information. However, it should be mentioned that present study had some limitations owing to the cross-sectional design prevent any causal conclusion between diet, CRF, and metabolic syndrome being distinguished. Also, Residual disruptors that are unmeasurable and possible in observational studies could affect the study. Finally, it is important to note that the findings of this study may not be generalize to beyond this sample of adults.

## Conclusion

Overall, adherence to identified dietary patterns in this population was associated with some components of MetS. However, nor CRF neither the combination of dietary patterns and CRF was related to the odds of MetS among Iranian adults. More studies are needed to clarify these associations and to consider environmental and interpersonal determinants. However, due to the cross-sectional design of the present study, more longitudinal studies are needed for more accurate conclusions.

## Supplementary Information

**Additional file 1: Supplementary figure 1**. Scree plot of 25 food groups according ti its eigenvalues

## Data Availability

The datasets generated or analyzed during the current study are not publicly available but are available from the corresponding author on reasonable request.

## Abbreviations

HDP: Healthy Dietary Pattern
MDP: Mixed Dietary Pattern
WDP: Western Dietary Pattern
CRF: Cardiorespiratory Fitness
FBS: Fasting Blood Sugar
TC: Total Cholesterol
TG: Triglyceride
HDL-C: High Density Lipoprotein
LDL-C: Low Density Lipoprotein
SBP: Systolic Blood Pressure
DBP: Diastolic Blood Pressure
FFM: Fat Free Mass
FM: Fat Mass
WC: Waist Circumference
CVD: cardiovascular disease
WHR: waist to hip ratio
BMI: Body Mass Index
