# Supplementary figures and images for: Interaction between major dietary patterns and cardiorespiratory fitness on metabolic syndrome in Iranian adults: a cross-sectional study
Source: Nutr J. 2021 Apr 13;20:36. doi: 10.1186/s12937-021-00695-4 (PMC8045397; doi:10.1186/s12937-021-00695-4)

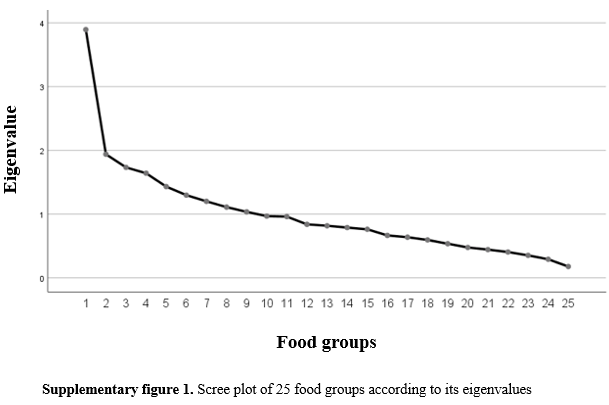

Supplement: Supplementary file 1 — Additional file 1: Supplementary figure 1. Scree plot of 25 food groups according ti its eigenvalues [file 12937_2021_695_MOESM1_ESM.tif]
